# Physicochemical properties of edible cricket oils: Implications for use in pharmaceutical and food industries

**DOI:** 10.1016/j.fufo.2024.100316

**Published:** 2024-06

**Authors:** Dorothy K. Murugu, Arnold N. Onyango, Alex K. Ndiritu, Dorothy N. Nyangena, Isaac M. Osuga, Xavier Cheseto, Sevgan Subramanian, Sunday Ekesi, Chrysantus M. Tanga

**Affiliations:** a International Centre of Insect Physiology and Ecology (*icipe*), P.O. Box 30772-00100 Nairobi, Kenya; b Department of Human Nutrition Sciences, Jomo Kenyatta University of Agriculture and Technology, P.O. Box 62000-00200 Nairobi, Kenya; c Department of Animal Science, Jomo Kenyatta University of Agriculture and Technology, P.O. Box 62000-00200 Nairobi, Kenya; d Department of Public Health, University of Kabianga, P.O. Box 2030-20200 Kericho, Kenya

**Keywords:** Edible insects, Cricket oil, Oil stability, shelf life, Nutraceuticals, Novel food ingredients

## Abstract

The prevailing global market demands locally produced, sustainable oils for biomedical applications. This study focused on evaluating the quality of cricket-derived oils and meals from *Scapsipedus icipe* Hugel, Tanga, and *Gryllus bimaculatus* De Geer common delicacy in Africa, following standard methods for physicochemical properties, fatty acid composition, and phytochemicals (oxalates, phytates, tannins, and polyphenols). The cricket oils physicochemical properties aligned with Codex Alimentarius standards for edible oils, including low solidification temperature (< 2 °C), a high refractive index (1.46), and a specific gravity of 0.88. Notably, peroxide values (1.9 to 2.5 mg mEq O2/kg), acid values (1.1 to 2.2 mg KOH/g), and saponification values (234–246 mg KOH/g) all are indicative of lightness and unsaturated fatty acids. Nutritionally, cricket powder was rich in protein (56.8–56.9% -) and fat (31.7–33.5% -of dry matter), with significant amounts of essential omega-3 and omega-6 fatty acids. Predominant saturated and monounsaturated fatty acids were palmitic (23.9–31.2 mg/100 g-) and oleic acids (10.9–11.4 mg/100 g- of oil), respectively. Antioxidant values (48.0 to 65.0 mg/100 g), inferred from total polyphenols, suggests a stable oil with long shelf-life. These results highlight the promising and sustainable potential of cricket-derived oils for applications in the food and pharmaceutical industries.

## Graphical abstract

**Figure:**
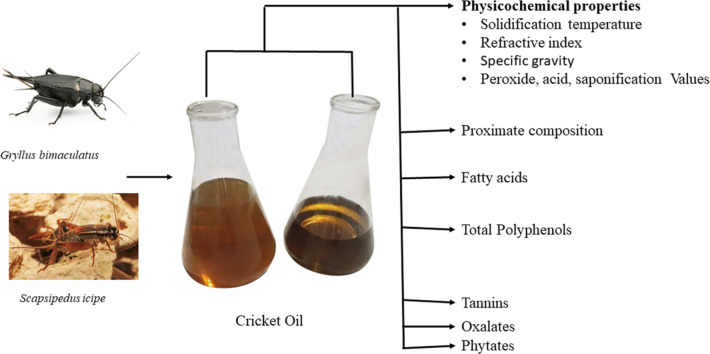

## 1 Introduction

Edible insects have long been incorporated into human diets across different regions worldwide, contributing significantly to improved livelihoods (van Huis et al., 2013) and a multitude of health benefits (Baiyegunhi et al., 2016). These insects are rich sources of essential nutrients, such as high-quality protein, fats, minerals, vitamins, as well as trace elements like phytosterols, alkaloids, and flavonoids (Baiyegunhi et al., 2016; Cheseto et al., 2015; Durst et al., 2010; van Huis et al., 2013). As a result, there has been a parallel surge in the commercialization of edible insects on a global scale (Agbidye et al., 2009; Mmari et al., 2017).

Among these diverse edible insect options, crickets emerge as a particularly promising species, largely due to their high protein content and potential for large-scale production. Consequently, there has been a growing interest in exploring the applications of cricket oils in various industries, including food and pharmaceuticals (Skotnicka et al., 2021). This burgeoning interest in the utilization of cricket-derived resources is poised to reshape various sectors and holds significant promise for sustainable, nutrient-rich alternatives in our ever-evolving world.

While crickets are increasingly valued for their high protein content, their lipid content, which includes beneficial antioxidants and omega-3 & 6 fatty acids, remains underexplored (Tzompa-Sosa et al., 2021).The lipid content in crickets varies significantly, ranging from 22% to 32%, with dietary composition, developmental stage, and sex all exerting influence (Ghosh et al., 2017; Magara et al., 2021); notably, female crickets tend to have higher oil content than males. (Kulma et al., 2019). Cricket oils have shown promise in lowering the risk of coronary heart disease (Blomquist et al., 1991; Oonincx and Finke, 2020) thanks to their high content of Omega-3 and Omega-6 fatty acids, which can also inhibit the production of inflammation-inducing prostaglandin hormones (Tzompa-Sosa et al., 2021). Furthermore, crickets are an excellent source of vitamin E, renowned for its anti-inflammatory properties (Murugu et al., 2021; Simopoulos, 2010, 2002).

However, due to their high unsaturated fatty acid (comprising more than two-thirds of the total oil) content, cricket oils are susceptible to deterioration (Tzompa-Sosa et al., 2021). Fortunately, they are also rich in antioxidants, which can enhance the oil's quality (Ugur et al., 2021). The presence and effectiveness of these antioxidants may vary depending on the species, sex, life stage, and diet of the crickets (Magara et al., 2021). Moreover, crickets contain phytochemicals such as phenolic compounds and phytosterols, which exhibit potential defense properties, nutritional and pharmacological benefits (Jimenez-Garcia et al., 2018; Opitz and Müller, 2009; Tzompa-Sosa et al., 2021). Nonetheless, crickets also contain anti-nutrients that can hinder protein digestibility and mineral availability although these challenges can be addressed through appropriate processing methods (Khattab and Arntfield, 2009; Ojha et al., 2021). Jimenez-Garcia et al. (2018)

The increasing use of cricket oils in food applications is rapidly gaining attention and momentum, primarily due to their unique properties and promising potential in a wide array of culinary uses. These insect-derived oils have shown remarkable versatility, finding application in various food products such as salad dressings, mayonnaise, baked goods, and confectionery items (Brynning et al., 2020; Phuah et al., 2023). Their exceptional emulsifying properties, attributed to the presence of phospholipids, with lecithin being a key component, facilitate the stabilization of oil and water mixtures, thereby enhancing the texture and extending the shelf life of baked goods (Bueschelberger et al., 2015; Olaleye et al., 2023). This multifaceted utility aligns with the surging demand for evolving insect-based ingredients, where insect oils emerge as a sustainable and nutrient-rich game-changer in the food industry. It harmonizes with shifting consumer preferences for nontoxic, healthier, and more eco-friendly products while promoting cleaner and transparent food labels (Shine, 2020).

In this study, we aimed to bridge the gap in our understanding of cricket meals and oils due to the scarcity of available data. Our objective was to provide valuable insights thorough analysis of the proximate composition of powder meal from two cricket species. Additionally, we evaluated their oil's physicochemical properties, fatty acid composition, and selected anti-nutrient profiles.

## 2 Materials and methods

### 2.1 Sample collection and preparation

Crickets were procured from nearby farms within the Nyanza region of Kenya. Sampled crickets were generally fed a diet comprising homemade composite flour (whole maize, soybean, and amaranth grain), agricultural side streams such as waste vegetable leaves (kales, sweet potato leaves, pumpkin leaves), and ripe banana peels. About 5 kg sample of each cricket sample (*G. bimaculatus* and *S. icipe*) were placed into standard nonwoven bags (12 × 16 inches). The live samples were frozen at −21 °C using a chest freezer, packed with ice-packs into cool boxes and transported to the International center of Insect Physiology and Ecology (*icipe*) laboratories for taxonomic identification using developed toolkits (Tanga et al., 2018).

The insect samples were separately weighed into composite portions each weighing 500 g. After thawing, these portions were ground and placed in 20 cm × 14.5 cm × 8 cm plastic Tupperware containers (Kenpoly, Nairobi, Kenya). Subsequently, the containers were stored in a deep freezer at-21 °C until ready to undergo analysis for both oil quality and phytochemical composition (Cricket samples were maintained at freezing temperatures for a duration of 48 h prior to the commencement of the oil extraction process).

### 2.2 Proximate composition of processed cricket meal

Proximate analyses were conducted following the approved methods outlined by the Association of Official Analytical Chemists (AOAC, 1990). Moisture and dry matter were determined by measuring loss after oven drying the sample at 105 °C for 3 h. The estimation of crude protein (CP) was conducted using the Kjeldahl method. Initially, the samples underwent digestion in concentrated Sulfuric acid, followed by titration in an automatic Kjeldahl analyzer (Velp UDK 159, Velp Scientifica, Europe). To determine the CP value, a nitrogen-to-protein conversion factor of 6.25 was applied. (Finke, 2007). The determination of crude fat utilized the Soxhlet extraction method, employing petroleum ether as the extractant. This was carried out in a Soxhlet extractor (Velp SER 148, Velp Scientifica, Europe). The quantification of crude ash involved a gravimetric approach using a muffle furnace maintained at 550 °C for a duration of 3 h. For the assessment of crude fiber, acid digestion followed by loss on ignition was conducted using a fiber analyzer (FIWE, Velp Scientifica, Europe).

### 2.3 Extraction of cricket oil

**Fig. 1:**
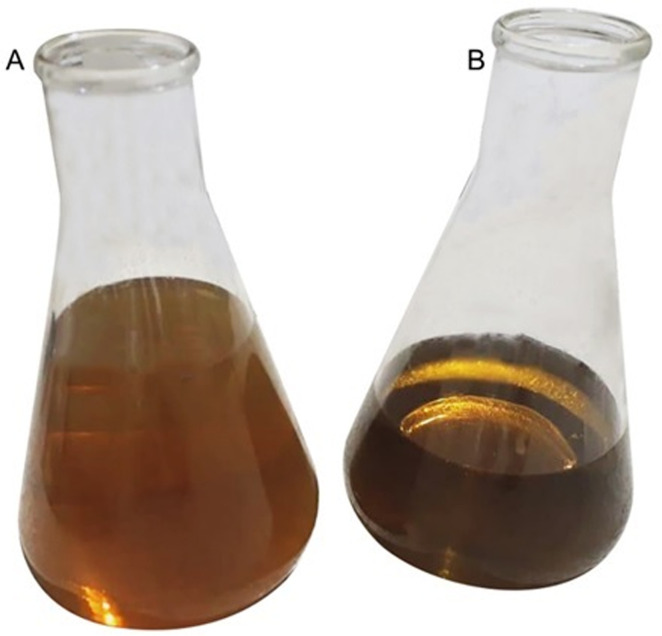
Oils extracted from *Scapsipedus icipe* (A) and *Gryllus bimaculatus* (B).

Cricket oil was extracted following the modified Folch's method (Igiehon et al., 2021). Five grams of milled samples was transferred into a conical flask containing 50 mL (chloroform – methanol, 2:1 v/v, with 10 mg/L butylated hydroxytoluene). The mixture underwent vortexing for 10 s, then sonication for 10 min and was subsequently left undisturbed for 30 min before centrifugation (1500 g, 23 °C) for 5 min. The resulting supernatant was then carefully transferred into a separating funnel where it was combined with 20 mL of 0.9% NaCl solution. After vigorous shaking, the mixture was allowed to settle until a biphasic system became evident. The upper aqueous phase was then discarded. The lower phase was dried over anhydrous Na_2_SO_4_ into pre-weighed conical flasks (Fig. 1). The solvent was removed in vacuo, and the percentage (%) of the total extracted oils calculated utilizing Eq. (1). The physicochemical properties of the oil were evaluated on the same day after extraction to ensure the preservation of sample integrity. Notably, the fat content was based on proximate values reported in a previous study (Murugu et al., 2021).

(1) $$\frac{\left[\left(weight\;of\;conical\;flask+oil\right)-\left(the\;weight\;of\;the\;conical\;flask\right)\right]}{\left(initial\;weight\;of\;the\;sample\right)\;}$$

### 2.4 Determination of physicochemical properties of cricket oil

#### 2.4.1 Determination of solidification temperature

The solidification temperature of the cricket oil was determined following the constant cooling rate approach, in accordance with the ASTM standard test method (Kruka et al., 1995). The oil was carefully introduced into a test tube, filling it halfway, alongside a mercury-bulb glass thermometer positioned at the test tube's base. Subsequently, the test tube was positioned on a gasket in a temperature-controlled cooling bath. At intervals of 1 °C reduction in temperature, the test tube was raised from the gasket, examined for any sign of cloudiness, and swiftly placed back into the cooling bath. The point at which solid fat formations became visible prompted the notation of the thermometer's reading as the definitive solidification temperature for the cricket the oil. This experiment was repeated three times using different batches of cricket oil samples.

#### 2.4.2 Determination of refractive index

The refractive index was assessed using an Abbe's refractometer in accordance with the procedure outlined in method 921.08 (AOAC, 2002). To eliminate impurities, insect oil samples (1 mL) were filtered through a Whatman filter paper no.1. The refractometer's prism was meticulously cleaned, dried and set to a temperature of 40.0 ± 0.1 °C. A small amount of insect sample (two- drops) was gently positioned on the lower prism. Upon securing the prism tightly with the screw head, the sample was left undisturbed for a duration of 2 min. Subsequently, adjustments were made to the refractometer lighting conditions to achieve a clear reading, which was then documented as the refractive index of the insect oil. This procedure was conducted in triplicate, employing distinct batches of the insect oil samples.

#### 2.4.3 Determination of specific gravity

The determination of specific gravity (SG) was conducted following the procedure outlined in method 920.212 (AOAC, 1998) using a 25 mL specific gravity bottle. Prior to filling it with insect oil, the empty specific gravity bottle was calibrated and its initial weight (W1) recorded. Subsequently, the bottled was filled with insect oil up to the designated mark, and its weight after filling (W2) was noted. In a similar manner, the specific gravity bottle was filled with water and its weight (W3) recorded. To ensure accuracy, this process was replicated three times using distinct batches of insect oil. The specific gravity of the oils was then determined using Eq. (2).

(2) $$SG=\frac{\left(W2-W1\right)}{\left(W3-W1\right)}$$

#### 2.4.4 Determination of peroxide, acid, and saponification values

The determination of peroxide (PV), acid (AV), and saponification values (SV) was carried out using established AOAC protocols: -AOAC method No. 965.33, AOAC method No. 920.160 and AOAC method No. 920.160 respectively (AOAC, 1998). To assess the peroxide value (PV), a sample weighing 2.5 g of insect oil was measured and placed into a glass stoppered flask. Subsequently, a mixture of glacial acetic acid and chloroform in a 3:2 v/v ratio (25 mL), along with saturated potassium iodide (1 mL), was added to the flask. After gentle agitation for 1 min, the solution was kept in darkness for 30 min. Water (30 mL) was then added and the flask shaken for 30 s. Two drops of starch solution were added and the mixture titrated against sodium thiosulphate of 0.01 mol/L concentration until the color changed from blue to colorless (end point). A parallel blank was concurrently prepared and analyzed. The calculation of PV (mEq O_2_/kg) was performed using Eq. (3).

(3) $$\left(PV=\;\left(V_{S}-V_{B}\right)\times N\times \;1000\right)/W$$

Where:

*V_S_* = sodium thiosulphate (mL) titrated against the oil sample.
*V_B_* = sodium thiosulphate (mL) titrated against the blank.
*N* = sodium thiosulphate concentration (mol/L).
*W* = oil sample weight (g).

To determine the acid value (AV), 2 g of insect oil sample was weighed into a 250 mL conical flask. Subsequently, 50 mL of neutralized ethyl alcohol was introduced to the flask. The resulting mixture was boiled in a water bath maintained at 80 °C for 5 min. Afterward, titration against 0.1 mol/L potassium hydroxide with phenolphthalein as the indicator. The calculation of the oil's AV in milligrams of potassium hydroxide (mg KOH/g) was performed using Eq. (4).

(4) (AV=A×M×56.1)/W

Where:

*A* = potassium hydroxide (mL) used.
*M* = KOH concentration (mol/L).
*W* = oil sample weight (g).

To determine the saponification value (SV), 2 g of insect oil was weighed into an Erlenmeyer flask. 25 mL of alcoholic potassium hydroxide (0.5 mol/L) was then added and the mixture was boiled under a reflux condenser for 30 min. The mixture was then cooled and titrated against hydrochloric acid (0.5 mol/L) using phenolphthalein as the indicator. A blank was similarly prepared, analyzed and SV (mg KOH/g) calculated using Eq. (5).

(5) $$\left(SV=\left(V_{B}-V_{S}\right)\times N\times \;28.05\right)/W$$

Where:

*V_B_* = hydrochloric acid (mL) titrated against the blank.
*V_s_* = hydrochloric acid (mL) titrated against the oil sample.
*N* = hydrochloric acid concentration (mol/L).
28.05 = the equivalent molecular weight of potassium hydroxide (0.5 mol/L).
*W* = oil sample weight (g).

### 2.5 Coupled gas chromatography–mass spectrometry (GC–MS) analysis of fatty acids

Gas chromatography-mass spectrometry (GC–MS) was utilized for the analysis of the fatty acid composition, employing methyl esterification method previously described (Cheseto. et al., 2020). A portion of the insect oil sample (0.1 g) was combined with sodium methoxide in methanol (0.5 mL) at a concentration of 15 mg/mL, vortexed for 10 s, sonicated for 5 min and then incubated at 60 °C for 1 h. Following this, deionized water (100 μL) was introduced to the mixture and vortexed for 1 min. To extract the resulting methyl esters, a 1 mL n-hexane (GC-grade) was introduced to the sample, followed by centrifugation at 14,000 rpm for 5 min. The supernatant was then dried using anhydrous sodium sulfate before being transferred into 2 mL clear glass GC-vials. The injection of methyl esters (1 µL) into a GC–MS system was carried out using an auto sampler 7683 (Agilent Technologies, Inc., Beijing, China) operating in the splitless mode. The GC–MS comprised a 7890A gas chromatograph (Agilent Technologies, Inc., Santa Clara, CA, USA) equipped with a 5975 C mass selective detector (Agilent Technologies, Inc., Santa Clara, CA, USA). During analysis, the inlet temperature of the GC was maintained at 270 °C, while the transfer line temperature was set to 280 °C. For the column oven, the temperature was programmed to rise from 35 °C to 285 °C. The initial temperature of 35 °C was held for 5 min, followed by an increment of 10 °C per minute until reaching 280 °C. This temperature was maintained for 20.4 min. A low bleed capillary column ((5%-phenyl)-methylpolysiloxane, (HP5 MS)) with dimensions of 30 m length × 0.25 mm internal diameter, and 0.25 μm film thickness) (J&W, Folson, CA, USA), was employed, utilizing helium as the carrier gas with a flow rate of 1.25 mL/min. The mass selective detector was sustained at quadrupole (180 °C) and ion source (230 °C) temperatures. Electron impact (EI) mass spectra were acquired with an acceleration energy of 70 eV, scanning fragment ions over a mass range of 40–550 *m/z* in full scan mode. A 3.3-min solvent delay time for the filament was set. Identification of fatty acids was achieved by comparing their methyl ester fragmentation patterns and retention times with known fatty acid methyl ester standards where available, and tentatively, from reference spectra present in library–MS databases such as the National Institute of Standards and Technology (NIST) 05, 08, and 11. To establish a linear calibration curve, standard methyl octadecenoate (Sigma-Aldrich, St. Louis, MO) with a purity of ≥ 95% and known concentrations (0.2, 5, 25, 50, 75, 100, and 125 ng/μL) was subjected to the same GC–MS conditions. The resulting curve (*y* = 7E + 06x – 4E + 07, R^2^ = 0.9757) was employed for external quantification of fatty acid methyl esters. The quantities of fatty acid methyl esters were expressed both in mg/100 g of oil and as a percentage.

### 2.6 Determination of phytochemical composition

#### 2.6.1 Determination of oxalic acid content

The oxalic acid content was assessed using high performance liquid chromatography (HPLC) (SDD/m20A, Shimadzu, Kyoto, Japan) as previously described (Libert and Franceschi, 1987) with modifications by (Yu et al., 2002). In brief, 0.5 g sample of ground insects was mixed with 10 mL of 0.5 mol/L hydrochloric acid and heated for 10 min at 80 °C. The mixture was allowed to cool to room temperature and 25 mL of distilled water was added, centrifuged at 10,000 rpm for 10 min at 25 °C, filtered and an aliquot (20 µL) injected to the HPLC system equipped with a photodiode array detector set at 210 nm and a C-18 column (250 mm length x 4.6 mm internal diameter, and 5.0 μm particle size, ACE, Advance Chromatography Technologies, Aberdeen, Scotland). Sulfuric acid (0.01 mol/L) was used as the mobile phase. The oxalic acid content was quantified by extrapolation from a linear calibration curve obtained from known oxalic acid standard concentrations (10–100 µg/mL).

#### 2.6.2 Determination of phytic acid content

The phytic acid content in the insect sample was analyzed using HPLC based on a method described earlier by (Camire and Clydesdale, 1982) with slight modification. First, 5 g of the blended insect sample was mixed with 3% sulfuric acid and subsequently, phytic acid precipitated using iron (III) chloride, centrifuged at 2500 rpm for 10 min after which the supernatant was discarded. The precipitate was washed with 30 mL of distilled water, and subsequent to another round of centrifugation under identical conditions, the supernatant was then discarded. The iron (III) chloride -phytate complex was converted to sodium phytate by adding 3 mL of sodium hydroxide (1.5 mol/L) and sonicating for 5 min. The volume of the mixture was adjusted to 30 mL with distilled water and then boiled for 30 min to precipitate iron (III) hydroxide. The cooled sample was centrifuged and the supernatant was quantitatively transferred to a 50 mL volumetric flask. The precipitate was rinsed with 10 mL of distilled water and the supernatant was added to the volumetric flask. The volume was made up to 50 mL with distilled water. A 0.45 µm syringe microfilter was used to filter the sample. A 20 µL aliquot of the filtered sample was injected into an HPLC system equipped with an octadecylsilyl C-18 column (250 × 4.6 mm x 5.0 μm) and a refractive index detector. Potassium dihydrogen phosphate (0.025 mol/L) was used as the mobile phase. The phytic acid content was quantified by extrapolation from a linear calibration curve obtained from standardized sodium phytate of the concentration range from 50 to 1000 µg/mL.

#### 2.6.3 Determination of tannin content

The tannin content was analyzed using the modified vanillin-hydrochloric acid method with catechin (5 mg/mL; Sigma-Aldrich Chemie, Steinheim, Germany) being used as the standard (Price and Butler, 1977). First, 250 mg of blended insect sample was mixed with 4% hydrochloric acid in methanol (10 mL). The mixture was shaken for 20 min and centrifuged at 4500 rpm for 10 min. The supernatant was quantitatively transferred into a 25 mL volumetric flask. The residue was extracted again with 1% hydrochloric acid in methanol (5 mL). The second supernatant was combined with the first supernatant and diluted to 25 mL. Seven concentrations (0–100 µg/mL) of standard catechin were also prepared. One mL of the extract and standard aliquot were each transferred to a test tube that contained 5 mL freshly prepared vanillin-hydrochloric acid reagent (equal volumes of 8% hydrochloric acid in methanol and vanillin in methanol). The mixture was left undisturbed for 20 min and then the absorbance was measured in a 1-cm glass cell at 500 nm using a spectrophotometer (Zeiss PMQ I1). The tannin content was expressed as percent catechin equivalent from a linear calibration curve generated.

#### 2.6.4 Determination of total polyphenols

The total polyphenol content of the insect sample was analyzed using the Folin–Ciocalteu method (Cong-Cong et al., 2017). First, 10 g of the blended insect sample was mixed with 50% aqueous methanol (20 mL) at 80 °C for 1 h. The solution was then filtered and the volume was made to 50 mL. A portion of the solution (1 mL) was transferred to a volumetric flask (50 mL) and the following reagents were added: 20 mL of water, 2.5 mL of Folin–Ciocalteu reagent, and 10 mL of 17% sodium carbonate. The mixture was homogenized and then the volume adjusted to 50 mL with distilled water. The solution was left undisturbed for 20 min and then the absorbance was measured in a 1 cm glass cell at 765 nm using a UV–visible spectrophotometer (SP65, Gallenkamp, UK). The total polyphenol content was calculated as gallic acid equivalents (GE) from a linear calibration curve generated.

### 2.7 Data analysis

To ascertain disparities in the proximate composition, physical-chemical properties, fatty acid constituents, and phytochemical makeup of the oils derived from the two distinct cricket species, the unpaired *t*-test was used for data that met the assumptions of normality and equal variances. In cases where the given assumptions were not met, Welch's *t*-test was utilized. The statistical analysis was performed using R software, version 4.0.5 (R Core Team and R Development Core Team, 2018).

## 3 Results

### 3.1 Proximate composition of cricket powder

The analysis comparing the nutritional composition of the two cricket species are presented (Table 1). The data show that both crude protein and fiber content of *G. bimaculatus* and *S. icipe* powder did not vary significantly. However, *G. bimaculatus* had significantly higher crude fat content (33.5%) compared to *S. icipe* (31.7%). Similarly, *G. bimaculatus* had a higher crude ash content (5.4%) than *S. icipe* (5.3%). Overall, the energy content per 100 g of cricket species was high, with *G. bimaculatus* having a slightly higher value of 529.2 kcal/100 g compared to *S. icipe* (512.66 kcal/100 g).

**Table 1:** Proximate composition of processed powder of *Gryllus bimaculatus* and *Scapsipedus icipe*.

Cricket Species	Crude protein [%]	Crude fat [%]	Crude ash [%]	Crude fiber [%]	Energy (Kcal/100 g)
*Scapsipedus icipe*	56.80 ± 0.40^a^	31.74 ± 0.25^a^	5.25 ± 0.02^a^	5.71 ± 1.39^a^	512.66
*Gryllus bimaculatus*	56.90 ± 1.33^a^	33.51 ± 0.16^b^	5.41± 0.06^b^	8.39 ± 2.28^a^	529.2
P- value	0.9092	0.0005	0.0123	0.1564	

### 3.2 Physicochemical properties of cricket oil

The physicochemical properties of oils from *G. bimaculatus* and *S. icipe* are described in Table 2. The differences recorded for refractive index and specific gravity were not statistically significant. The peroxide (PV) and saponification values (SV) of oil from *G. bimaculatus* and *S. icipe* were not significantly different. The AV of oil from *S. icipe* was 2-folds higher than that recorded for *G. bimaculatus* (Table 2).

**Table 2:** Physicochemical characteristics of *Gryllus bimaculatus* and *Scapsipedus icipe* oil.

Parameter	Insect species		t value	*P*-value
	*G. bimaculatus*	*S. icipe*		
Solidification temperature (°C)	2 – 7	2 – 5		
Refractive index	1.46 ± 0.02^a^	1.47 ± 0.02^a^	−2.598	0.1047
Specific gravity	0.88 ± 0.01^a^	0.89 ± 0.06^a^	2.36	0.0774
Acid value (mg KOH/g)	1.10 ± 0.01^a^	2.19 ± 0.36^b^	180.29	< 0.001
Peroxide value (mEq O_2_/kg)	1.92 ± 0.45^a^	2.49 ± 0.95^a^	−22.89	0.3581
Saponification value (mg KOH/g)	246 ± 3.02^a^	234 ± 9.51^a^	2.064	0.108

In the same row, means (±standard deviations) with the same superscript letters are not significantly different at *P* ≥ 0.05.

### 3.3 Composition of fatty acids in cricket oils

**Fig. 2:**
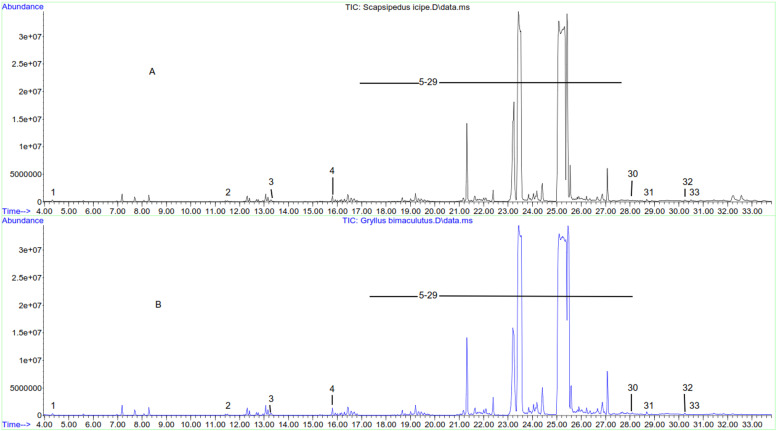
Representative overlaid total ion chromatogram of the fatty acid profile. *A* = *Scapsipedus icipe* and *B* = *Gryllus bimaculatus*. Numbers 1–33 fatty acid identified as shown in Table 3.

The fatty acid profiles of *S. icipe* and *G. bimaculatus* are presented in Fig. 2 and Table 3. The fatty acid groups followed the order: SFA > MUFA > PUFA for *G. bimaculatus*, while that of *S. icipe* followed the order: SFA > PUFA >MUFA. Palmitic acid and stearic acids were predominantly higher compared to other saturated fatty acids (SFA) in *S. icipe* and *G. bimaculatus* accounting for 81 and 80%, respectively. Caprylic and pentadecanoic acids were not detected in *G. bimaculatus*.

**Table 3:** Composition of fatty acids (% of total fatty acids *, (mg/100 g) *) of *Scapsipedus icipe* and *Gryllus bimaculatus*.

Peak No.	tR (min)	Fatty Acid Methyl Ester	Fatty acid	Insect species		t value	P-value
				*S. icipe*	*G. bimaculatus*		
1^a^	4.56	Methyl butanoate	Butanoic acid (C4:0)	0.001±0.0^a^ (0.2)	0.004±0.0^b^ (0.54)	−6.298	0.001
2 a	11.23	Methyl heptanoate	Enanthic acid (C7:0)	0.09±0.01^a^ (14.46)	0.21±0.03^b^ (31.72)	−6.641	0.001
3 a	13.67	Methyl octanoate	Caprylic acid (C8:0)	0.06±0.02^a^ (9.93)	Nd	7.250	0.001
4 a	15.80	Methyl decanoate	Capric acid (C10:0)	0.04±0.04^a^ (5.76)	0.02±0.01^a^ (3.34)	1.617	0.09
5 a	17.23	Methyl undecanoate	Undecanoic acid (C11:0)	0.26±0.02^a^ (40.81)	0.35±0.1^a^ (52.69)	−1.479	0.106
6 a	18.98	Methyl dodecanoate	Lauric acid (C12:0)	0.31±0.04^a^ (48.48)	0.35±0.02^a^ (51.94)	−1.341	0.125
7 a	20.07	Methyl tridecanoate	Tridecylic acid (C13:0)	0.22±0.06^a^ (33.83)	0.42±0.07^b^ (62.83)	−3.643	0.010
11 a	21.32	Methyl tetradecanoate	Myristic acid (C14:0)	5.43±0.85^b^ (841.53)	3.7 ± 0.58^a^ (554.17)	2.926	0.021
12 a	22.37	Methyl pentadecanoate	Pentadecanoic acid (C15:0)	0.08±0.03^a^ (12.03)	Nd	4.643	0.004
15 a	23.66	Methyl hexadecanoate	Palmitic acid (C16:0)	23.89±0.63^a^ (3699.45)	31.18±0.7^b^ (4668.27)	−13.406	<0.001
16 a	24.05	Methyl heptadecanoate	Margaric acid (C17:0)	1.44±0.1^a^ (222.85)	1.4 ± 0.27^a^ (210.5)	0.206	0.423
19 a	25.55	Methyl octadecanoate	Stearic acid (C18:0)	17.26±1.56^b^ (2670.53)	14.71±1.36^a^ (2200.85)	2.138	0.049
20 a	26.21	Methyl nonadecanoate	Nonadecanoic acid (C19:0)	0.73±0.13^a^ (113.23)	0.83±0.13^a^ (123.93)	−0.92	0.204
28 a	27.07	Methyl eicosanoate	Arachidic acid (C20:0)	0.004±0.0^a^ (0.64)	0.01±0.0^b^ (1.41)	−2.229	0.044
29 b	27.87	Methyl heneicosanoate	Heneicosylic acid (C21:0)	0.5 ± 0.07^b^ (76.66)	0.32±0.08^a^ (47.37)	2.827	0.023
31 b	28.66	Methyl docosanoate	Behenic acid (C22:0)	0.37±0.03^b^ (757.61)	0.01±0.0^a^ (1.34)	20.073	<0.001
33 b	30.21	Methyl tetracosanoate	Lignoceric acid (C24:0)	0.28±0.02^a^ (43.98)	0.26±0.03^a^ (38.56)	1.239	0.141
			∑SFA (%)	50.96	57.60		
8 b	20.10	Methyl (9*Z*)-decenoate	Caproleic acid (C10:1)	Nd	0.14±0.02^a^ (21)	−10.341	<0.001
9 b	21.01	Methyl (9*Z*)-dodecenoate	Lauroleic acid (C12:1 n-3)	0.16±0.02^a^ (24.56)	0.12±0.04^b^ (18.19)	1.443	0.111
10 a	21.15	Methyl (9*Z*)-tetradecenoate	Myristoleic acid (C14:1)	Nd	0.09±0.01^a^ (13.37)	−15.678	<0.001
13 b	23.24	Methyl (9*Z*)-hexadecenoate	Palmitoleic acid (C16:1)	11.14±0.97^b^ (1727.07)	9.22±0.63^a^ (1382.17)	2.869	0.022
18 a	25.24	Methyl (9*Z*)-Octadecenoate	Oleic acid (C18:1 n-9)	11.4 ± 0.81^a^ (1767.2)	10.94±0.81^a^ (1639.54)	0.69	0.263
26 b	26.86	Methyl (11*Z*)-eicosenoate	Gondoic acid (C20:1 n-9)	1.56±0.25^b^ (241.3)	0.86±0.18^a^ (128.92)	3.968	0.008
27 b	26.99	Methyl (13*Z*)-eicosenoate	Paullinic acid (C20:1 n-7)	0.01±0.0^a^ (0.92)	0.01±0.0^a^ (1)	−1.314	0.129
32 b	30.03	Methyl (13*Z*)-tetracosenoate	Nervonic acid (C24:1 n-9)	0.05±0.01^a^ (7.71)	0.29±0.06^b^ (43.34)	−6.467	0.001
			**∑MUFA (%)**	**24.33**	**23.24**		
14 b	23.55	Methyl (7Z,10Z,13Z)-hexadecatrienoate	Hexadecatrienoic acid (HTA) (C16:3 n-3)	Nd	0.01±0.0^a^ (0.91)	−12.901	<0.001
17 a	25.21	Methyl (9Z,12Z)-octadecadienoate	Linoleic acid (C18:2 n-6)	23.56±0.25^a^ (3647.9)	23.34±0.51^a^ (3493.35)	0.673	0.268
21 a	26.34	Methyl(9Z,12Z,15Z)-octadecatrienoate	Linolenic acid (C18:3 n-3)	0.21±0.1^a^ (32.1)	0.32±0.04^a^ (48.6)	−1.823	0.071
22 b	26.35	Methyl (5Z,11Z,14Z)-eicosatrienoate	Sciadonic acid (C20:3 n-6)	0.86±0.09^a^ (133.18)	0.79±0.04^a^ (118.49)	1.192	0.149
23 b	26.40	Methyl (5Z,8Z,11Z,14Z-eicosatetraenoate	Arachidonic acid (C20:4 n-6)	Nd	0.01±0.0^a^ (0.95)	−8.123	<0.001
24 b	26.45	Methyl (6Z,9Z,12Z,15Z-octadecatetraenoate	Eicosatetraenoic acid (ETA) (C20:4 n-3)	Nd	0.01±0.0^a^ (1.12)	−12.422	<0.001
25 a	26.54	Methyl(5Z,8Z,11Z,14Z,17Z)-eicosapentaenoate	Eicosapentaenoic acid (EPA) (C20:5 n-3)	0.08±0.0^a^ (12.17)	0.07±0.0^a^ (10.97)	1.524	0.101
30 a	28.12	Methyl (4Z,7Z,10Z,13Z,16Z,19Z)-docosahexaenoate	Docosahexaenoic acid (DHA) (C22:6 n-3)	0.01±0.0^a^ (1.7)	0.01±0.0^a^ (1.87)	−0.534	0.31
			**∑PUFA (%)**	**24.71**	**19.16**		
			**∑PUFA: ∑SFA**	**0.48**	**0.33**		
			**∑n-6/∑n-3**	**53.09**	**44.70**		

Peak No. = Peak Number.

a = fatty acid identity confirmed with authentic standard.

b = fatty acid tentatively identified; tR (min) = Retention time (minutes); * = Mean ± standard deviations of triplicate determinations. In the same row, means (±standard deviations) with the same superscript letters are not significantly different at *P* ≥ 0.05. *Values in parenthesis are fatty acid values (mg/100 g oil sample). SFA= saturated fatty acids; MUFA= monounsaturated fatty acids; PUFA= polyunsaturated fatty acids; ∑n-6= sum of linoleic, sciadonic and arachidonic acids; ∑n-3= sum of lauroleic, linolenic, ETA, EPA and DHA acids.

The total monounsaturated fatty acid (MUFA) content in *S. icipe* was higher than in *G. bimaculatus* by 1.04 folds. Oleic acid was the most predominant MUFA in both cricket species. Butenoic and myristoleic acids were not detected in *S. icipe*. The total polyunsaturated fatty acid (PUFA) content in *S. icipe* was 1.3 folds higher than that of *G. bimaculatus*. Linoleic acid represented more than two thirds of total PUFA in both cricket species.

## 4 Phytochemical properties of cricket oils

The phytochemical composition of *G. bimaculatus* and *S. icipe* are presented in Table 4. Oxalates did not vary significantly between the two cricket species. The phytate concentration differed significantly between *G. bimaculatus* and *S. icipe*. However, total polyphenols in *G. bimaculatus* were significantly lower compared to *S. icipe*.

**Table 4:** Phytochemical composition (mg/100 g) of *Gryllus bimaculatus* and *Scapsipedus icipe*.

	Insect species		t value	*P*-value
	*G. bimaculatus*	*S. icipe*		
Oxalates	97.01 ± 9.14^a^	75.13 ± 5.94^a^	−1.993	0.076
Phytates	10.33 ± 6.00^b^	3.85 ± 0.44^a^	−3.23	0.012
Tannins^1^	1.56 ± 1.08^a^	Nd*	−4.342	0.003
Total polyphenols^2^	48.00 ± 4.49^a^	65.00 ± 1.79^b^	2.797	0.021

In the same row, means (±standard deviations) with the same superscript letters are not significantly different at *P* ≥ 0.05. Nd*= Not detected.

1 *Catechin equivalents*.

2 *Gallic acid equivalents*.

## 4 Discussion

Fat is the second largest proximate component of edible insects after crude protein, which is consistent with our current findings for *G. bimaculatus* and *S. icipe*. In this study, fat content in the two crickets ranged between 32 and 34%, similar to that reported by (Murugu et al., 2021). The quantities reported here are not exhaustive and are varied based on the fat extraction method used and physiological status of the insects (Laroche et al., 2019; Psarianos et al., 2022).

The solidification temperature values (2–7 °C for *G. bimaculutus* and 2–5 °C for *S.icipe*) presented in this study were lower than the values reported for termites (*Macrotermes subhylanus* Rambur) (8–12 °C) and grasshoppers (*R. differens*) (10–15 °C) (Kinyuru, 2021). This low solidification temperature is an indication that the cricket oils are fluids at room temperature (Ekpo et al., 2009).

The refractive index values for oil extracted from the cricket species (1.460 ± 0.02 for *G. bimaculutus* and 1.470 ± 0.02 for *S.icipe*) were similar to that from edible caterpillar (*Imbrasia oyemensis* Rougeot) (1.4675 ± 0.0002), (Patrice et al., 2015) This is in agreement with refractive index values for groundnut (1.464 ± 0.002) and acacia oils (*A. colei* 1.473 ± 0.001; *A. tumida* 1.474 ± 0.000) (Falade et al., 2008). The high refractive index seems to confirm presence of unsaturated fatty acids or presence of long chain fatty acids (Eromosele and Paschal, 2003). Interestingly, the refractive index of both cricket species closely mirrors that of common vegetable oils, as outlined in the Codex Alimentarius for fats and oils (Table 5) (FAO/WHO, 2001)

**Table 5:** Refractive index of common vegetable oils.

Type of oil	Refractive index
*S. icipe* oil	1.440 - 1.480
*G. bimaculutus* oil	1.450 - 1.490
Peanut oil	1.460 - 1.465
Maize oil	1.465 - 1.468
Mustard oil	1.461 - 1.469
Sesame oil	1.465 - 1.469
Soybean oil	1.466 - 1.470
Sunflower oil	1.461 - 1.468
Palm oil	1.458 - 1.460

Source: Codex Alimentarius: Fats, Oils and Related Products –(FAO/WHO, 2001).

The specific gravity of the two cricket oils were comparable to the values reported elsewhere (*Macrotermes bellicosus*, 0.90 ± 0.01) (Ekpo et al., 2009), but less dense than that reported for edible caterpillar (0.942 ± 0.02), (*I. oyemensis*) (Patrice et al., 2015), termites (0.93 ± 0.01), (*M. subhylanus*) and grasshoppers (*R. differens*) (0.94 ± 0.02), (Kinyuru, 2021) and conventional edible oils (Conophor oil 0.912±0.912; Raw linseed oil 0.931±0.936) (Akpuaka and Nwankwor, 2000). Therefore, oils from *G. bimaculatus* and *S. icipe* could be considered lighter than conventional oils and hence be useful in high value industries like pharmaceuticals.

The chemical properties of the oils such as saponification (SV), peroxide (PV) and acid values (AV) often indicate the stability of the oils during processing and storage (Guillén and Cabo, 2002). The differences in chemical properties are largely dependent on the fatty acid profile, which in turn depends on the insect species, sex, developmental stage and diet (Kinyuru et al., 2013; Lehtovaara et al., 2017; Oonincx et al., 2015). The AV of *G. bimaculatus* (1.10 ± 0.01 mg KOH/ g) and *S. icipe* (2.19 ± 0.36 mg KOH/ g) were similar but higher than that reported for palm weevil larvae (*Rhynchophorus phoenicis* Fabricius) (1.62 ± 0.31 mg KOH/ g) (Tiencheu et al., 2013). However, the AV for both crickets were lower than the values reported for rhinoceros beetle larvae (*O. owariensis*) (3.73 ± 0.02 mg KOH/ g) (Assielou et al., 2016) and the recommended value for Codex Alimentarius standard for a virgin oil (4.0 mgKOH/ g) (Codex Alimentarius, 2019).

Both *S. icipe* and *G. bimaculatus* had 2.5–19.1- and 2–14.7-times higher PV, respectively, than that reported for rhinoceros beetle larvae (*O. owariensis*) (0.96 ± 0.02 mEq O_2_/ kg) (Assielou et al., 2016), termites (*M. subhylanus*) (0.19 ± 0.01 mEq O_2_/ kg) and grasshoppers (*R. differens*) (0.14 ± 0.02 mEq O_2_/ kg) (Kinyuru et al., 2010). On the other hand, these cricket species had lower PV compared to commercial oils like olive oil (8 mEq O_2_/kg) and palm oil (8 mEq O_2_/kg) (Azlan et al., 2010).

The PV of the cricket species was below the Codex general standards for good oils (10 mEq O_2_/kg) (Codex Alimentarius, 2019). The low PV and AV found in the cricket species could be attributed to the existence of antioxidants such as α-tocopherol (Kinyuru et al., 2010; Murugu et al., 2021). This implies that oils from both cricket species would be less susceptible to oxidation during processing and storage (Ekop et al., 2010; Musundire et al., 2014) and thus could be utilized in food processing industries.

The Saponification Value (SV) serves as a metric for determining the average molecular weight of the fatty acids within an oil. A heightened SV corresponds to a reduced average molecular weight of the fatty acids, while a lower SV signifies the opposite. In the realm of the food industry, the SV of edible oils emerges as a crucial indicator of oil quality. An elevated SV suggests a prevalence of short- and medium-chain fatty acids in the oil, characteristics generally deemed more favorable from a nutritional standpoint (Tiencheu et al., 2021). The saponification values for *G. bimaculatus* and *S. icipe*, at 246 mg KOH/g and 243 mg KOH/g, respectively, surpassed those of certain other cricket species such as *Brachytrupes membranaceus* (180 mg KOH/g) (Tiencheu et al., 2021) and *Acheta domesticus* (216.5 mg KOH/g), (Kamau et al., 2017). Notably, these values were found to be comparable to the saponification values of coconut oil (>250 mg KOH/g) (FAO/WHO, 2001), a well-known and widely used oil according to FAO/WHO (2001). This suggests that *G. bimaculatus* and *S. icipe* oils exhibit saponification values that are particularly noteworthy, aligning closely with the properties of a recognized and commonly utilized oil like coconut oil.

Fatty acid profiles in edible insects largely depends on diets, which explains why the palmitic and stearic acid were recorded as the most predominant SFAs in *G. bimaculatus* and *S. icipe* (Opitz and Müller, 2009). *Scapsipedus icipe* had similar values for palmitic acid as those reported elsewhere by (Starčević et al., 2017). However, palmitic values reported for *G. bimaculatus* were higher by 1.3 folds to that reported by (Starčević et al., 2017). Stearic acid values were consistently higher than those reported elsewhere by (Starčević et al., 2017; Yang et al., 2006). On the contrary, both cricket species had considerably higher SFA values than that recorded for the dagaa fish (39.4%) and Nile tilapia (34.2%) when compared to whole milk (67.4%) as demonstrated in the Kenya food composition table (Codex Alimentarius, 2019; FAO and Kenya, 2019; Yang et al., 2006).

Oleic acid was the most predominant MUFA in both crickets, which is consistent with the findings reported for other cricket species (FAO and Kenya, 2019; Starčević et al., 2017; Yang et al., 2006). However, these values for both cricket species were lower compared to that recorded for the field crickets (*Gryllus testaceus* Walker and *Gryllus assimilis* Fabricius) (FAO and Kenya, 2019; Starčević et al., 2017). The considerable variation might be attributed to feed and geographical differences. Previous studies on crickets and termites have reported that palmitoleic acid was the second most abundant MUFA (Starčević et al., 2017).

Oleic acid has been reported to have modulatory effects in health and disease. For instance, (Sales-Campos et al., 2013) demonstrated the potential role of oleic acid in enhancing immune function by modulating leucocytes, reducing inflammation including its role in wound healing as well as in cancer prevention. Additionally, a review of intervention studies confirmed the role of oleic acid in reducing cardiovascular risk when added to milk as a substitute to counter the effects of saturated fatty acids with direct implications in lowering cholesterol levels (Lopez-Huertas, 2010).

Linoleic acid as the predominant PUFA was in agreement with findings reported for other cricket species (Starčević et al., 2017; Yang et al., 2006). The linoleic acid value reported here are within the range of values reported for crickets elsewhere (Yang et al., 2006). Nonetheless, *G. bimaculatus* and *S. icipe* showed lower linoleic acid values compared to that reported for other crickets such as *Homorocoryphus nitidulus* Scopoli (Womeni et al., 2009) and Jamaica field crickets *G. assimilis* fed on different oil blend diets (Starčević et al., 2017). The presence of essential fatty acids - arachidonic, eicosatetraenoic, eicosapentaenoic and docosahexaenoic acids in *G. bimaculatus* and *S. icipe* could contribute to key roles in children's growth and development when incorporated in their daily diets through conventional foods (Wainwright, 1992). In addition, the presence of eicosenoic acids (gondoic and paullinic acid) in both cricket species could lead to their potential utilization in the cosmetic industry. All the differences observed in the MUFA and PUFA content in the crickets could be attributed to the variation of diet composition (Kinyuru et al., 2010; Starčević et al., 2017; Womeni et al., 2009). Both crickets had less PUFA content compared to dagaa fish (36.22%) and Nile tilapia (31.97%) as reported in the Kenya food composition table (FAO and Kenya, 2019).

Nutritionally, the consumption of foods with high contents of SFA is undesirable due to their association with high risk of cardiovascular diseases. This is contrary to the intake of unsaturated fatty acids (UFA), known to reduce the prevalence of cardiovascular diseases (Simopoulos, 2004). This therefore implies that *G. bimaculatus* and *S. icipe* are nutritionally superior in terms of quality due to higher PUFA/SFA ratios compared to that derived from beef and whole milk (FAO and Kenya, 2019). However, PUFA/SFA ratios in both crickets are lower compared to that from Nile tilapia and dagaa fish (FAO and Kenya, 2019). Higher ratio of omega 6 (n-6) to omega 3 (n-3) fatty acids has also been deemed undesirable, as it is linked to high risk of immunological disorders (Wainwright, 1992). According to (Gogus and Smith, 2010), a joint report from FAO and WHO recommended a ratio of n-6 to n-3 of <5:1, while Canada recommended a range (4:1 to 10:1) for a balanced food. The cricket species studied had ratios of n-6 to n-3 that were within the ranges (4:1 to 55:1) recommended (Starčević et al., 2017; Yang et al., 2006). This ratio is largely dependent on the insect's diet (Starčević et al., 2017), which implies that diet manipulation could lead to the production of cricket products that have maximum positive impact on human health.

**Fig. 3:**
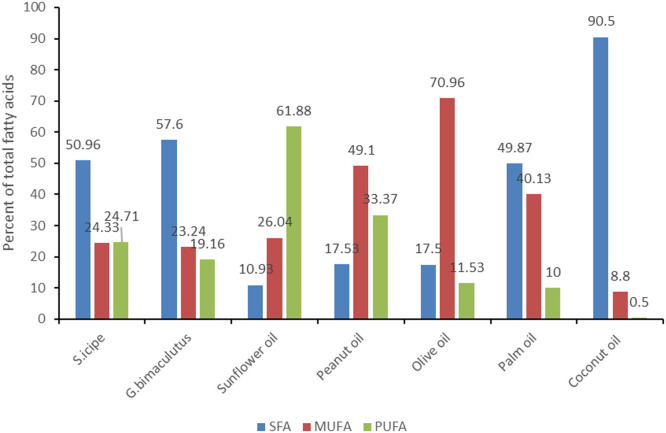
Comparison of fatty acid composition in popular vegetable edible oils and crickets *S. icipe* and *G. bimaculutus.*

In this study, we conducted a comprehensive analysis of the fatty acid composition of two cricket oils and compared it with that of other reported food sources (Fig. 3). The comparison demonstrated that cricket oil exhibits a composition that aligns favorably with common well-established vegetable oils like sunflower oil (Harun, 2019), peanut oil (Giuffrè et al., 2016), olive oil (Giuffrè, 2010), palm oil (Japir et al., 2017), and coconut oil (Rohman et al., 2021)

Our analysis reported several noteworthy fatty acids present in the cricket's sample, such as Omega-3 fatty acids (eicosapentaenoic acid, EPA, and docosahexaenoic acid, DHA), Omega-6 fatty acids (linoleic acid, LA, and γ-linolenic acid, GLA), and monounsaturated fatty acids (oleic acid). In the context of pharmaceutical use, Omega-3 fatty acids, particularly EPA and DHA, have demonstrated anti-inflammatory and neuroprotective properties, suggesting their potential in the treatment of neurological disorders (Holub, 2002; Mori, 2017; Simopoulos, 2002; Swanson et al., 2012). Moreover, the presence of GLA has been associated with anti-inflammatory effects and may find applications in conditions related to inflammation (González-Fernández et al., 2020; Li et al., 2020; Sinclair et al., 2003).

From an industrial standpoint, these fatty acids can have diverse applications. For instance, oleic acid, a monounsaturated fatty acid, is commonly used in the production of cosmetics, soaps, and pharmaceuticals (Almeida et al., 2006; Bengal, 2013; Ekpo et al., 2009; Foodcircle, 2023). Additionally, Omega-3 fatty acids are sought after for their use in nutritional supplements and functional foods (Cencic and Chingwaru, 2010), contributing to both the pharmaceutical and health industries. Unfortunately, the pharmaceutical industry has yet to fully capitalize on the untapped potential of cricket oil.

This underutilization can be attributed to several factors, including the pharmaceutical industry's limited awareness of cricket oil as an ingredient and the regulatory hurdles associated with introducing new pharmaceutical products to the market (Dossey et al., 2016; Stull and Patz, 2020). Nevertheless, there is a noticeable surge in interest among pharmaceutical companies regarding cricket oil. As research on the health benefits of cricket oil continues to expand, coupled with an increasing focus on product development, it is foreseeable that cricket oil will gain broader acceptance and integration into the pharmaceutical industry.

Phytochemicals such as oxalates, phytates and tannins are considered anti-nutrients as they chelate minerals like calcium and magnesium consequently making them unavailable for absorption and utilization in the body (Udousoro et al., 2018). In addition, oxalates could lead to formation of calcium oxalate complexes in kidneys hence, kidney stones (Kunatsa et al., 2020). Although tannins are considered anti-nutrients, they have been shown to have both biological and pharmacological activity such as anti-oxidative, antibacterial, antiviral, anti-inflammatory, immune-modulatory and cardio-protective (Kumari and Jain, 2012). The values reported in this study for oxalates and tannins were lower, while those of phytates were higher compared to those reported for crickets (*Henicus whellani* Chopard) (Kumari and Jain, 2012; Kunatsa et al., 2020).

The oxalates and phytate concentration presented for the edible crickets studied were higher than values reported earlier for four edible insects consumed in Nigeria: crickets (*Gymnogryllus lucens* Walker), yam bettles (*Heteroligus meles* Billberg), palm weevils (*R. phoenicis*) and grasshoppers (*Zonocerus variegatus* Linnaeus) (Ekpo et al., 2009). However, only *G. bimaculatus* had higher tannin levels compared to values reported for *G. lucens* (Ekpo et al., 2009; Omotoso and Adesola, 2018) reported tannin level (0.54–1.13 mg/100 g) in four edible insects while (Chakravorty et al., 2016) reported values (496.67–615 mg/100 g) in two edible insects in India. The tannins values reported in edible insects in India were higher compared to that obtained for *G. bimaculatus* and *S. icipe*. With respect to recommended permissible levels (oxalates: 250 mg/100 g; phytates: 250–500 mg/100 g) (Ekop et al., 2010; Musundire et al., 2014), both crickets in this study have insignificant levels of anti-nutrients.

Total polyphenols are used to indicate the antioxidant capacity of a food (Viuda-Martos et al., 2011). In the study of (Liu et al., 2012), polyphenols were demonstrated to inhibit lipid oxidation in black chafer beetle (*Holotrichia parallela* Motschulsky) extracts. Therefore, the presence of polyphenols in *G. bimaculatus* and *S. icipe* oils could contribute to their preservation during their application as food ingredients. The cricket species in this study had about 0.01–0.06 times lower polyphenols compared to edible insects reported elsewhere including crickets (*Henicus whellani* and *A. domesticus*), mealworms (*Tenebrio molitor*), superworms (*Zophobas morio* Fabricius), and palm weevils (*Rhynchophorus ferrugineus* Olivier) (Botella-Martínez et al., 2021). However, the polyphenol concentrations reported for *G. bimaculatus* and *S. icipe* were about 5 times higher than the value reported in other studies for termites (*Macrotermes facilger*) (Kunatsa et al., 2020).

In China, the use of insects as antioxidants have been associated with the treatment of diseases linked to oxidative stress such as immunodeficiency, cancer and heart disease (Liu et al., 2012). (Liu et al., 2012) further demonstrated the ability to use insects and insect products as nutraceuticals in alleviating oxidative induced diseases as well as being natural antioxidant additives in the food industry. The variability of phytochemicals in edible insects is reliant on the species type, gut emptying by starvation prior to harvesting, portion of insect used for extract, methods and solvents used in the extraction of phytochemicals (Botella-Martínez et al., 2021). Processing techniques of insects into value-added products to reduce anti-nutrient factors have received limited research attention (Imathiu, 2020), although some traditional cooking methods like boiling have revealed remarkable reduction in anti-nutrient level in few foods (Palermo et al., 2014)

## 5 Conclusion

In conclusion, cricket oils presented in this study have demonstrated great potential for utilization in food and pharmaceutical industry based on their physicochemical characteristics. These oils are less susceptible to rancidity as exhibited by their low peroxide and acid values. These implies cricket oils are a healthier option for incorporation into human and animal diets based on higher MUFAs and lower SFAs values when compared to conventional animal and plant sources. Our findings have shown that the presence of bioactive compounds in cricket oils offer consumers huge opportunities related to protection from many chronic diseases. Scaling the health-promoting properties of both cricket species, would require their integration into market-driven consumer appealing and familiar food products. However, effective processing methods to significantly reduce anti-nutrient concentrations to safer levels would be crucial.

## Ethical statement - studies in humans and animals

No studies on animals were conducted in this research work. This research was approved by the Institutional Animal Care and Use Committee (IACUC) of Kenya Agricultural and Livestock Research Organization (KALRO)-Veterinary Science Research Institute (VSRI); Muguga North upon compliance with all provisions vetted under and coded: KALRO-VSRI/IACUC028/16032022.

## CRediT authorship contribution statement

**Dorothy K. Murugu:** Data curation, Formal analysis, Investigation, Methodology, Project administration, Validation, Visualization, Writing – original draft, Writing – review & editing. **Arnold N. Onyango:** Formal analysis, Project administration, Supervision, Validation, Writing – review & editing. **Alex K. Ndiritu:** Data curation, Formal analysis, Supervision, Writing – original draft. **Dorothy N. Nyangena:** Data curation, Supervision, Validation, Writing – original draft. **Isaac M. Osuga:** Supervision, Validation, Writing – review & editing. **Xavier Cheseto:** Conceptualization, Data curation, Formal analysis, Investigation, Methodology, Supervision, Writing – original draft, Writing – review & editing. **Sevgan Subramanian:** Conceptualization, Formal analysis, Funding acquisition, Project administration, Resources, Supervision, Writing – review & editing. **Sunday Ekesi:** Funding acquisition, Project administration, Supervision, Writing – review & editing. **Chrysantus M. Tanga:** Conceptualization, Funding acquisition, Project administration, Resources, Supervision, Validation, Writing – review & editing.

## Declaration of competing interest

The authors declare that they have no conflict of interest. The funders had no role in the design of the study, in the collection, analyses or interpretation of data, in the writing of the manuscript or in the decision to publish the results.

## Data Availability

All the supporting data used in the manuscript has been submitted to a public repository (https://www.pangaea.de/tok/8ab034849495ca1e0108296cf15e2d0ee242aaf5).

## References

[bib0001] Agbidye F.S., Ofuya T.I., Akindele S.O. (2009). Marketability and nutritional qualities of some edible forest insects in Benue State, Nigeria. Nigeria. Pak J Nutr..

[bib0002] Akpuaka M.U., Nwankwor E. (2000). Extraction, analysis and utilization of a drying-oil from *Tetracarpidium conophorum*. Bioresour. Technol..

[bib0003] Almeida J.C.de, Perassolo M.S., Camargo J.L., Bragagnolo N., Gross J.L. (2006). Fatty acid composition and cholesterol content of beef and chicken meat in Southern Brazil. Braz. J. Pharm. Sci..

[bib0006] AOAC (1990).

[bib0005] AOAC (1998).

[bib0004] AOAC, 2002. Measuring the Refractive Index of Oils and Fats | AOAC 921.08 [WWW Document]. Association of Official Analysis Chemists International. URL https://www.mt.com/ph/en/home/library/applications/lab-analytical-instruments/refractive-index-of-oils-and-fats-aoac-92108.html (accessed 7.14.23).

[bib0007] Assielou B., DUE E.A., Koffi D.M., Kouame P. (2016). Physicochemical characterization and fatty acid composition of *Oryctes owariensis* larvae oil. Food Env. Saf..

[bib0008] Azlan A., Prasad K.N., Khoo H.E., Abdul-Aziz N., Mohamad A., Ismail A., Amom Z. (2010). Comparison of fatty acids, vitamin E and physicochemical properties of *Canarium odontophyllum* Miq. (dabai), olive and palm oils. J. Food Compost. Anal..

[bib0009] Baiyegunhi L.J.S., Oppong B.B., Senyolo G.M. (2016). Mopane worm (*Imbrasia belina*) and rural household food security in Limpopo province. South Africa. Food Secur..

[bib0010] Bengal W. (2013). Assessment of nutritional quality and anti-nutrient composition of two edible grasshoppers (Orthoptera: acrididae) - a Search for New Food Alternative. IJMPS.

[bib0011] Blomquist G.J., Borgeson C.E., Vundla M. (1991). Polyunsaturated fatty acids and eicosanoids in insects. Insect Biochem..

[bib0012] Botella-Martínez C., Lucas-González R., Pérez-Álvarez J.A., Fernández-López J., Viuda-Martos M. (2021). Assessment of chemical composition and antioxidant properties of defatted flours obtained from several edible insects. Food Sci. Technol. Int..

[bib0013] Brynning G., Bækgaard J.U., Heckmann L.H.L. (2020). Investigation of consumer acceptance of foods containing insects and development of non-snack insect-based foods. Ind. Biotechnol..

[bib0014] Bueschelberger H.G., Tirok S., Stoffels I., Schoeppe A. (2015).

[bib0015] Camire A.L., Clydesdale F.M. (1982). Analysis of phytic acid in foods by HPLC. J. Food Sci..

[bib0016] Cencic A., Chingwaru W. (2010). The role of functional foods, nutraceuticals, and food supplements in intestinal health. Nutrients..

[bib0017] Chakravorty J., Ghosh S., Megu K., Jung C., Meyer-Rochow V.B. (2016). Nutritional and anti-nutritional composition of *Oecophylla smaragdina* (Hymenoptera: formicidae) and *Odontotermes* sp. (Isoptera: termitidae): two preferred edible insects of Arunachal Pradesh. India. J. Asia Pac. Entomol..

[bib0019] Cheseto X., Kuate S.P., Tchouassi D.P., Ndung'u M., Teal P.E.A., Torto B. (2015). Potential of the desert locust *Schistocerca gregaria* (Orthoptera: acrididae) as an unconventional source of dietary and therapeutic sterols. PLoS. One.

[bib0018] Cheseto X., Baleba S.B.S., Tanga C.M., Kelemu S., Torto B. (2020). Chemistry and sensory characterization of a bakery product prepared with oils from African edible insects. Foods..

[bib0020] Codex Alimentarius (2019).

[bib0021] Cong-Cong X.U., Bing W., Yi-Qiong P.U., Jian-Sheng T.A.O., Zhang T. (2017). Advances in extraction and analysis of phenolic compounds from plant materials. Chin. J. Nat. Med..

[bib0022] Dossey A.T., Tatum J.T., McGill W.L., Dossey Aaron T, Morales-Ramos J.A., Rojas M.G. (2016). Insects As Sustainable Food Ingredients.

[bib0023] Durst P.B., Johnson D.V., Leslie R.N., Shono K. (2010). Proceedings of a workshop on Asia-Pacific resources and their potential for development.

[bib0024] Ekop E.A., Udoh A.I., Akpan P.E. (2010). Proximate and anti-nutrient composition of four edible insects in Akwa Ibom state. Nigeria. World J. Appl. Sci. Technol.

[bib0025] Ekpo K.E., Onigbinde A.O., Asia I.O. (2009). Pharmaceutical potentials of the oils of some popular insects consumed in southern Nigeria. Afr. J. Pharm. Pharmacol..

[bib0026] Eromosele C.O., Paschal N.H. (2003). Characterization and viscosity parameters of seed oils from wild plants. Bioresour. Technol..

[bib0027] Falade O.S., Adekunle A.S., Aderogba M.A., Atanda S.O., Harwood C., Adewusi S.R. (2008). Physicochemical properties, total phenol and tocopherol of some Acacia seed oils. J. Sci. Food Agric..

[bib0028] FAO, Kenya (2019).

[bib0029] FAO/WHO, 2001. Codex Alimentarius: fats, oils and related products, Codex alimentarius. FAO.

[bib0030] Finke M.D. (2007). Estimate of chitin in raw whole insects. Zoo Biol..

[bib0031] Foodcircle, 2023. Plant-based fats & oils in soaps [WWW Document]. URL https://www.foodcircle.com/magazine/plant-oils-fats-saponification-soaps (accessed 5.29.23).

[bib0032] Ghosh S., Lee S.M., Jung C., Meyer-Rochow V.B. (2017). Nutritional composition of five commercial edible insects in South Korea. J. Asia Pac. Entomol..

[bib0034] Giuffrè A.M., Tellah S., Capocasale M., Zappia C., Latati M., Badiani M., Ounane S.M. (2016). Seed oil from ten Algerian peanut landraces for edible use and biodiesel production. J. Oleo Sci..

[bib0035] Gogus U., Smith C. (2010). n-3 Omega fatty acids: a review of current knowledge. Int. J. Food Sci. Technol..

[bib0036] González-Fernández M.J., Ortea I., Guil-Guerrero J.L. (2020). α-Linolenic and γ-linolenic acids exercise differential antitumor effects on HT-29 human colorectal cancer cells. Toxicol. Res..

[bib0037] Guillén M.D., Cabo N. (2002). Fourier transform infrared spectra data versus peroxide and anisidine values to determine oxidative stability of edible oils. Food Chem..

[bib0038] Harun M. (2019). Fatty acid composition of sunflower in 31 inbreed and 28 hybrid. Biomed. J. Sci. Technol. Res..

[bib0039] Holub B.J. (2002). Clinical nutrition: 4. Omega-3 fatty acids in cardiovascular care. CMAJ..

[bib0040] Igiehon N.O., Babalola O.O., Cheseto X., Torto B. (2021). Effects of rhizobia and arbuscular mycorrhizal fungi on yield, size distribution and fatty acid of soybean seeds grown under drought stress. Microbiol. Res..

[bib0041] Imathiu S. (2020). Benefits and food safety concerns associated with consumption of edible insects. NFS J..

[bib0042] Japir A.A.W., Salimon J., Derawi D., Bahadi M., Al-Shuja'A S., Yusop M.R. (2017). Physicochemical characteristics of high free fatty acid crude palm oil. OCL.

[bib0043] Jimenez-Garcia S.N., Vazquez-Cruz M.A., Garcia-Mier L., Contreras-Medina L.M., Guevara-González R.G., Garcia-Trejo J.F., Feregrino-Perez A.A. (2018). Phytochemical and pharmacological properties of secondary metabolites in berries. Therapeutic Foods.

[bib0044] Kamau E., Mutungi C., Kinyuru J., Imathiu S., Tanga C., Affognon H., Ekesi S., Nakimbugwe D., Fiaboe K.K.M. (2017). Effect of packaging material, storage temperature and duration on the quality of semi-processed adult house cricket meal. J. Food Res..

[bib0045] Khattab R.Y., Arntfield S.D. (2009). Nutritional quality of legume seeds as affected by some physical treatments 2. Antinutritional factors. LWT.

[bib0047] Kinyuru J.N., Kenji G.M., Njoroge S.M., Ayieko M. (2010). Effect of processing methods on the in vitro protein digestibility and vitamin content of edible winged termite (*Macrotermes subhylanus*) and grasshopper (*Ruspolia differens*). Food Bioproc. Tech..

[bib0048] Kinyuru J.N., Konyole S.O., Roos N., Onyango C.A., Owino V.O., Owuor B.O., Estambale B.B., Friis H., Aagaard-hansen J., Kenji G.M. (2013). Nutrient composition of four species of winged termites consumed in western Kenya. J. Food Compos..

[bib0046] Kinyuru J.N. (2021). Oil characteristics and influence of heat processing on fatty acid profile of wild harvested termite (*Macrotermes subhylanus*) and long-horned grasshopper (*Ruspolia differens*). Int. J. Trop. Insect Sci..

[bib0049] Kruka V.R., Cadena E.R., Long T.E. (1995). Cloud-point determination for crude oils. J. Pet. Technol..

[bib0050] Kulma M., Kouřimská L., Plachý V., Božik M., Adámková A., Vrabec V. (2019). Effect of sex on the nutritional value of house cricket. *Acheta domestica* L. Food Chem..

[bib0051] Kumari M., Jain S. (2012). Tannins: an antinutrient with positive effect to manage diabetes. Res. J. Recent Sci..

[bib0052] Kunatsa Y., Chidewe C., Zvidzai C.J. (2020). Phytochemical and anti-nutrient composite from selected marginalized Zimbabwean edible insects and vegetables. J. Agric. Food Res..

[bib0053] Laroche M., Perreault V., Marciniak A., Gravel A., Chamberland J., Doyen A. (2019). Comparison of conventional and sustainable lipid extraction methods for the production of oil and protein isolate from edible insect meal. Foods..

[bib0054] Lehtovaara V.J., Valtonen A., Sorjonen J., Hiltunen M., Rutaro K., Malinga G.M., Nyeko P., Roininen H. (2017). The fatty acid contents of the edible grasshopper *Ruspolia differens* can be manipulated using artificial diets. J. Insects Food Feed..

[bib0055] Li G., Wang X., Yang H., Zhang P., Wu F., Li Y., Zhou Y., Zhang X., Ma H., Zhang W., Li J. (2020). α-Linolenic acid but not linolenic acid protects against hypertension: critical role of SIRT3 and autophagic flux. Cell Death. Dis..

[bib0056] Libert B., Franceschi V.R. (1987). Oxalate in crop plants. J. Agric. Food Chem..

[bib0057] Liu S., Sun J., Yu L., Zhang C., Bi J., Zhu F., Qu M., Yang Q. (2012). Antioxidant activity and phenolic compounds of *Holotrichia parallela* Motschulsky extracts. Food Chem..

[bib0058] Lopez-Huertas E. (2010). Health effects of oleic acid and long chain omega-3 fatty acids (EPA and DHA) enriched milks. A review of intervention studies. Pharmacol. Res..

[bib0033] Louadj L., Giuffrè A.M. (2010). Analytical characteristics of olive oil produced with three different processes in Algeria. Riv. Ital. Delle Sostanze Grasse..

[bib0059] Magara H.J.O., Niassy S., Ayieko M.A., Mukundamago M., Egonyu J.P., Tanga C.M., Kimathi E.K., Ongere J.O., Fiaboe K.K.M., Hugel S., Orinda M.A., Roos N., Ekesi S. (2021). Edible crickets (Orthoptera) around the world: distribution, nutritional value, and other benefits—a review. Front Nutr..

[bib0060] Mmari M.W., Kinyuru J.N., Laswai H.S., Okoth J.K. (2017). Traditions, beliefs and indigenous technologies in connection with the edible longhorn grasshopper *Ruspolia differens* (Serville 1838) in Tanzania. J. Ethnobiol..

[bib0061] Mori T.A. (2017). Marine omega-3 fatty acids in the prevention of cardiovascular disease. Fitoterapia.

[bib0062] Murugu D.K., Onyango A.N., Ndiritu A.K., Osuga I.M., Xavier C., Nakimbugwe D., Tanga C.M. (2021). From farm to fork: crickets as alternative source of protein, minerals, and vitamins. Front. Nutr..

[bib0063] Musundire R., Zvidzai C.J., Chidewe C., Samende B.K., Manditsera F.A. (2014). Nutrient and anti-nutrient composition of *Henicus whellani* (Orthoptera: stenopelmatidae), an edible ground cricket, in south-eastern Zimbabwe. Int. J. Trop. Insect Sci..

[bib0064] Ojha S., Bekhit A.E., Grune T., Schlu O.K. (2021). Bioavailability of nutrients from edible insects. Curr. Opin. Food Sci..

[bib0065] Olaleye A.A., Adeyeye E.I., Adesina A.J., Adubiaro H.O. (2023). Analytical evaluation of fatty acid, phospholipid and sterol profiles of five species of edible insects. Pak. J. Sci. Ind. Res..

[bib0066] Omotoso O.T., Adesola A.A. (2018). Comparative studies of the nutritional composition of some insect orders. Int. J. Entomol..

[bib0067] Oonincx D.G.A.B., Finke M.D. (2020). Nutritional value of insects and ways to manipulate their composition. J. Insects Food Feed..

[bib0068] Oonincx D.G.A.B., Van Broekhoven S., Van Huis A., Van Loon J.J.A. (2015). Feed conversion, survival and development, and composition of four insect species on diets composed of food by-products. PLoS. One.

[bib0069] Opitz S.E.W., Müller C. (2009). Plant chemistry and insect sequestration. Chemoecology..

[bib0070] Palermo M., Pellegrini N., Fogliano V. (2014). The effect of cooking on the phytochemical content of vegetables. J. Sci. Food Agric..

[bib0071] Patrice K., Amon Akpossan R., Due E.A., Koffi D.M., Kouame P. (2015). Fatty acids, mineral composition and physico-chemical parameters of imbrasia oyemensis larvae oil with unusual arachidonic acid content. J. Food Eng..

[bib0072] Phuah E.T., Lee Y.Y., Tang T.K., Lim S.A., Rambli M. (2023). Rheological, textural properties and storage stability of mayonnaise formulated with protein hydrolysate derived from yellow mealworm (*Tenebrio molitor*). J. Insects Food Feed..

[bib0073] Price M.L., Butler L.G. (1977). Rapid visual estimation and spectrophotometric determination of tannin content of sorghum grain. J. Agric. Food Chem..

[bib0074] Psarianos M., Dimopoulos G., Ojha S., Cavini A.C.M., Bußler S., Taoukis P., Schlüter O.K. (2022). Effect of pulsed electric fields on cricket (*Acheta domesticus*) flour: extraction yield (protein, fat and chitin) and techno-functional properties. IFSET.

[bib0075] R Core Team, R Development Core Team, 2018. A Language and environment for statistical computing. R Foundation for statistical computing.

[bib0076] Rohman A., Irnawati Erwanto Y., Lukitaningsih E., Rafi M., Fadzilah N.A., Windarsih A., Sulaiman A., Zakaria Z. (2021). Virgin coconut oil: extraction, physicochemical properties, biological activities and Its authentication analysis. Food Rev. Int..

[bib0077] Sales-Campos H., Reis de Souza P., Crema Peghini B., Santana da Silva J., Ribeiro Cardoso C. (2013). An overview of the modulatory effects of oleic acid in health and disease. Mini. Rev. Med. Chem..

[bib0078] Shine L. (2020).

[bib0081] Simopoulos A.P. (2002). Omega-3 fatty acids in inflammation and autoimmune diseases. J. Am. Coll. Nutr..

[bib0080] Simopoulos A.P. (2004). Omega-6/omega-3 essential fatty acid ratio and chronic diseases. Food Rev. Int..

[bib0079] Simopoulos A.P. (2010). The omega-6/omega-3 fatty acid ratio: health implications. Oléagineux, Corps gras, Lipides..

[bib0082] Sinclair A.J., Attar-Bashi N.M., Li D. (2003). What is the role of αt-linolenic acid for mammals?. Lipids.

[bib0083] Skotnicka M., Karwowska K., Kłobukowski F., Borkowska A., Pieszko M. (2021). Possibilities of the development of edible insect-based foods in Europe. Foods..

[bib0084] Starčević K., Gavrilović A., Gottstein Ž., Mašek T. (2017). Influence of substitution of sunflower oil by different oils on the growth, survival rate and fatty acid composition of Jamaican field cricket (*Gryllus assimilis*). Anim. Feed Sci. Technol..

[bib0085] Stull V., Patz J. (2020). Research and policy priorities for edible insects. Sustain. Sci..

[bib0086] Swanson D., Block R., Mousa S.A. (2012). Omega-3 fatty acids EPA and DHA: health benefits throughout life. Adv. Nutr..

[bib0087] Tanga C.M., Magara H.J.O., Ayieko M.A., Copeland R.S., Khamis F.M., Mohamed S.A., Ombura F.L.O., Niassy S., Subramanian S., Fiaboe K.K.M., Roos N., Ekesi S., Hugel S. (2018). A new edible cricket species from Africa of the genus *Scapsipedus*. Zootaxa.

[bib0089] Tiencheu B., Womeni H.M., Linder M., Mbiapo F.T., Villeneuve P., Fanni J., Parmentier M. (2013). Changes of lipids in insect (*Rhynchophorus phoenicis*) during cooking and storage. Eur. J. Lipid Sci. Technol..

[bib0088] Tiencheu B., Achidi A., Tenyang N., Ufuan Achidi A., Ngongang Eurydice Flore T., Namondo Mbongo Lyonga A., Dibanda Romelle F. (2021). Oils of *Rhynchophorusphoenicis* Larva and *Brachytrupes membranaceus*: chemical properties, fatty acid composition and its effects on serum lipid profile of Wistar albino rats. Int. J. Innov. Sci. Eng. Technol..

[bib0090] Tzompa-Sosa D.A., Dewettinck K., Provijn P., Brouwers J.F., de Meulenaer B., Oonincx D.G.A.B. (2021). Lipidome of cricket species used as food. Food Chem..

[bib0091] Udousoro I.I., Udo E.S., Udoh A.P., Udoanya E.E. (2018). Proximate and antinutrients compositions, and health risk assessment of toxic metals in some edible vegetables. NJCR..

[bib0092] Ugur A.E., Bolat B., Oztop M.H., Alpas H. (2021). Effects of high hydrostatic pressure (HHP) processing and temperature on physicochemical characterization of insect oils extracted from *Acheta domesticus* (house cricket) and *Tenebrio molitor* (yellow mealworm). Waste BioMass Valorization..

[bib0093] van Huis A., Van Itterbeeck J., Klunder H., Mertens E., Halloran A., Muir G., Vantomme P. (2013).

[bib0094] Viuda-Martos M., Ruiz-Navajas Y., Fernández-López J., Sendra E., Sayas-Barberá E., Pérez-Álvarez J.A. (2011). Antioxidant properties of pomegranate (*Punica granatum* L.) bagasses obtained as co-product in the juice extraction. Food Res. Int..

[bib0095] Wainwright P.E. (1992). Do essential fatty acids play a role in brain and behavioral development?. Neurosci. Biobehav. Rev..

[bib0096] Womeni H.M., Linder M., Tiencheu B., Mbiapo F.T., Villeneuve P., Fanni J., Parmentier M. (2009). Oils of insects and larvae consumed in Africa: potential sources of polyunsaturated fatty acids. Oléagineux, Corps gras. Lipides.

[bib0097] Yang L.F., Siriamornpun S., Li D. (2006). Polyunsaturated fatty acid content of edible insects in Thailand. J. Food Lipids..

[bib0098] Yu L., Peng X.X., Yang C., Liu Y.H., Fan Y.P. (2002). Determination of oxalic acid in plant tissue and root exudate by reversed phase high performance liquid chromatography. Chinese J. Anal. Chem..

